# Selected Extracts of Chinese Herbal Medicines: Their Effect on NF-**κ**B, PPAR**α** and PPAR**γ** and the Respective Bioactive Compounds

**DOI:** 10.1155/2012/983023

**Published:** 2012-05-23

**Authors:** E. Rozema, A. G. Atanasov, N. Fakhrudin, J. Singhuber, U. Namduang, E. H. Heiss, G. Reznicek, C. W. Huck, G. K. Bonn, V. M. Dirsch, B. Kopp

**Affiliations:** ^1^Department of Pharmacognosy, University of Vienna, Althanstrasse 14, 1090 Vienna, Austria; ^2^Department of Pharmaceutical Biology, Faculty of Pharmacy, Gadjah Mada University, Sekip Utara, Yogyakarta 55281, Indonesia; ^3^Institute of Analytical Chemistry and Radiochemistry, University of Innsbruck, Innrain 52a, 6020 Innsbruck, Austria

## Abstract

Chinese herbal medicinal (CHM) extracts from fourteen plants were investigated in cell-based *in vitro* assays for their effect on nuclear factor **κ**B (NF-**κ**B), a key regulator of inflammation, as well as on peroxisome proliferator-activated receptors (PPARs) being key regulators of genes involved in lipid and glucose metabolism. 43% of the investigated CHMs showed NF-**κ**B inhibitory and 50% PPAR**α** and PPAR**γ** activating effects. Apolar extracts from cortex and flos of *Albizia julibrissin* Durazz. and processed rhizomes of *Arisaema* sp. and *Pinellia ternata* (Thunb.) Breit. that effectively inhibited TNF-**α**-induced NF-**κ**B activation and dose-dependently activated PPAR**α** and PPAR**γ** were further investigated. Bioassay-guided fractionation and analysis by GC-MS led to the identification of fatty acids as PPAR agonists, including linoleic and palmitic acid.

## 1. Introduction

Herbal medicines are an important part of Traditional Chinese Medicine (TCM) of which the medical use and processing methods are well documented. Traditional processing methods (*pao zhi*) are important to enhance the efficacy and/or to reduce the toxicity of crude herbal products [1]. Chinese herbal medicine (CHM) encompasses over 11,000 species of medicinal plants and is a valuable source [2] for the identification of biologically active natural products. Investigation of the molecular targets and mechanistic action of CHMs and their single compounds is currently a central task in TCM research [3]. Thanks to advances in molecular biology, refined bioassays are now available which enable rapid screening of natural products for bioactivity towards specific targets [4]. In this study, extracts of different polarity from CHMs of fourteen plant species were tested for a potential inhibition of TNF-*α*-induced NF-*κ*B activation and an agonistic activity towards PPAR*α* and PPAR*γ*. The CHMs were selected in cooperation with Chinese partners [5]. This in-house collection of CHMs (Table 1) was already examined earlier based on their traditional use against insomnia and anxiety regarding a putative modulation of the GABA_A_ receptor [6]. The listed CHMs are, however, also traditionally prescribed as single herbs as well as in formulations for clearing* heat* and drying *dampness, *among others. Therefore, to gain insight into possible multi-target effects of the individual CHMs, this study examined their influence on the nuclear factor *κ*B (NF-*κ*B) pathway and peroxisome proliferator-activated receptors (PPARs), which are important drug targets with regard to inflammation and metabolic dysfunction.

The NF-*κ*B signaling pathway is a key regulator of inflammation. Inflammatory stimuli such as tumor necrosis factor alpha (TNF-*α*), infectious agents (lipopolysaccharide (LPS)), injury, and other stressful conditions activate the NF-*κ*B signal transduction pathway. Thereby, activation of the IKK complex leads to phosphorylation and subsequent proteasomal degradation of I*κ*B proteins. NF-*κ*B dimers are translocated to the nucleus and bind to *κ*B promotor sites resulting in the transcription of proinflammatory target genes [7, 8]. Inhibitors of NF-*κ*B signaling are promising candidates for both the prevention and therapy of (chronic) inflammation. Terpenoids, such as parthenolide, are known to be effective natural NF-*κ*B inhibitors [9].

PPARs regulate the expression of genes involved in lipid and glucose metabolism and homeostasis in a ligand-dependent manner [10]. In the activated form, PPAR*α* promotes mainly fatty acid (FA) catabolism, and PPAR*γ* enhances insulin sensitivity and lipid storage [11, 12]. PPAR*α* and PPAR*γ* agonism is also linked to the suppression of pro-inflammatory genes, for example, via an interference with the NF-*κ*B signaling pathway [13, 14]. Synthetic PPAR agonists (fibrates and thiazolidinediones) bind PPARs with high affinity. They, however, show significant dose limiting adverse side effects. Natural products were revealed to be a promising source of safer PPAR*α* and PPAR*γ* dual agonists or partial PPAR modulators [15–18].

In our study, extracts of selected CHMs that exhibited a significant activity towards PPARs or NF-*κ*B were further subjected to dose-response studies and bioassay-guided fractionation. These selected CHMs were cortex and flos of *Albizia julibrissin *Durazz. as well as processed and crude rhizomes of *Arisaema *sp. and *Pinellia ternata *(Thunb.) Breit. Traditionally, *A. julibrissin *cortex and flos are used in CHM to treat insomnia and injuries and *to calm the mind*. The rhizomes of *Arisaema *sp. and *P. ternata *are toxic in their crude form and therefore traditionally processed (Table 1). Processed rhizomes of *Arisaema *sp. and *P. ternata* CHMs are effective in removing *damp-phlegm* such as in convulsions and spasms in the intestines [19, 20]. The dried, pulverized crude rhizomes from *P. ternata* are used externally to treat swellings. Bioactive compounds were isolated, analyzed, and characterized by mass spectrometry and NMR. 

## 2. Materials and Methods

### 2.1. Materials

Human embryonic kidney (HEK) 293 cells stably transfected with an NF-*κ*B-responsive luciferase reporter were purchased from Panomics (Fremont, CA, USA) and 3T3-L1 preadipocytes and HEK293 cells from the American Type Culture Collection (Manassas, VA, USA). The PPAR luciferase reporter construct (tk-PPREx3-luc) and the expression plasmids for PPAR*α* and PPAR*γ* (pCMX-mPPAR*α* and pCMX-mPPAR*γ*) were a gift from Professor Ronald M. Evans (Howard Hughes Medical Institute, La Jolla, CA, USA). The plasmid encoding enhanced green fluorescent proteins (pEGFP-N1) was obtained from Clontech (Mountain View, CA, USA). Dulbecco's modified Eagle's medium (DMEM) containing 4.5 g/L glucose was purchased from Lonza Group AG (Basel, Switzerland) and fetal bovine serum (FBS) from Invitrogen (Lofer, Austria). GW7647 and troglitazone were purchased from Cayman Europe (Tallinn, Estonia) and 2-deoxy-D-(1H^3^)-glucose from Perkin Elmer (Waltham, MA, USA). For extraction, fractionation, and isolation by silica column chromatography (CC), solvents of highest available purity were used (VWR, Vienna, Austria). All other chemicals were obtained from Sigma-Aldrich (Vienna, Austria).

### 2.2. Plant Materials

Material of the CHMs no. 1–14 were purchased from Plantasia (Oberndorf, Austria) (see Table 1) [19, 20], except of no. 12a which was purchased from Sichuan Neautus Traditional Chinese Medicine Co., Ltd (Chengdu, China). Voucher specimens are deposited at the Department of Pharmacognosy, University of Vienna, encoded with the CHM numbers shown in Table 1.

### 2.3. Extraction and Fractionation

50 g of each of the CHMs nos. 1a, 1b, 2a, 3–11, 12b, 13, and 14 was soaked in 500 mL petroleum ether (PE) for 10 min, and extracted under reflux for 30 min. The obtained extracts were filtered and evaporated to dryness. The remaining drug material was air-dried overnight and extracted each with 500 mL of ethyl acetate (EtOAc), methanol (MeOH), and distilled water likewise, whereas the water extract was lyophilized. All following extractions were performed at 150 bar and 40°C using an ASE 200 accelerated solvent extractor and a solvent controller (Dionex, Sunnyvale, CA, USA). With the ASE every ca. 10 g of pulverized plant material in a capsule was extracted three times with 22 mL of solvent (66 mL/ca. 10 g). The scheme of each run of extraction was as follows: 5 min heating, 2 min static at 150 bar and 40°C, 10 s flushing and 60 s purging. For CHMs nos. 2b, 12a, and 12b out of 50 g each DCM dry extract yields of 0.4–0.7% were obtained and for no. 1b a yield of 2.9% (See Schemes 1 and 2 in Supplementary Material available online at doi:10.1155/2012/983023).

For bioassay-guided fractionation, 1.4 kg of CHMs no. 1a and 1.9 kg of no. 2a were pulverized and extracted by DCM and subsequently by MeOH. 68 g DCM extract (4.7% yield) and 92 g MeOH extract (6.6% yield) were obtained of no. 1a. 12.1 g DCM (0.6% yield) and 10.7 g MeOH (0.8% yield), out of 1.4 kg of the material already extracted by DCM, were obtained of no. 2a. The dried extracts of no. 2a were subjected to silica CC with Silica Gel 60 (particle size of 0.063–0.200 mm, Merck, Darmstadt, Germany). The column with DCM extract was eluted with CHCl_3_ : MeOH : H_2_O 98 : 2 : 1 to 60 : 38 : 8.5 to obtain eight fractions (D1–D8). From fractions D8, cerebrosides were isolated by silica CC eluting with CHCl_3_ : MeOH : H_2_O 65 : 25 : 4. Cerebrosides were also detected in CHMs no. 12a and b from *P. ternata*. 15 g of DCM extract of no. 1a was fractionated using VLC (Vacuum Liquid Chromatography) using Silica Gel 60 (particle size of 0.063–0.200 mm, Merck, Darmstadt, Germany) as solid phase. The silica column was eluted subsequently with PE, DCM (twice), EtOAc, MeOH, and 90% MeOH (3 L per solvent) under reduced pressure. Thereby 12 mg PE extract, 963 mg extract from the first 3L eluted with DCM (DCM-I), 776 mg from the next 3L eluted with DCM (DCM-II), 6.90 g EtOAc extract, 4.08 g MeOH extract, and finally 170 mg extract eluted with 90% MeOH were obtained as dry extracts. Betulinic acid (12 mg) was isolated from EtOAc VLC fraction of the DCM extract from no. 1a. A mixture of trihydroxy and epoxy-dihydroxy fatty acid methyl esters (FAMEs) (8 mg) and *α*-spinasterol-3-*O*-*β*-D-glucopyranoside (61 mg) was isolated from the crude MeOH extract by repeated silica CC eluting with CHCl_3_ : MeOH : H_2_O and* n-*hexane : EtOAc gradients. The compounds in the mixture could be separated by GC-MS and were identified based on resemblance with the fragmentation in EI MS reported by Hamberg [21] as methyl 9,10,13-trihydroxy-11-octadecenoate, methyl 9,12,13-trihydroxy-11-octadecenoate, and methyl 9,10-epoxy-13-hydroxy-11-octadecenoate. Their hydroxylated FA derivatives were prepared by basic hydrolysis with 0.1 M NaOH (15 min RT) of the hydroxylated FAMEs and acidification with 0.1 M HCl/MeOH. For biological testing from all corresponding samples, always dry material was dissolved in DMSO.

### 2.4. NMR Analysis

All 1D (^1^H and ^13^C) and 2D (COSY, NOESY, HMQC, and HMBC) NMR spectra were recorded on a Bruker Avance 500 NMR spectrometer. Betulinic acid was dissolved in CDCl_3_ (99.96 atom% D) and *α*-spinasterol-3-*O*-*β*-D-glucopyranoside in d5-pyridine (99.96 atom% D). The ^1^H and ^13^C NMR spectra were operated at 500 and 125 MHz, respectively. ^13^C and ^1^H NMR data of both were consistent with the literature values for betulinic acid [22, 23] and *α*-spinasterol-3-*O*-*β*-D-glucopyranoside [24, 25].

### 2.5. Analysis of Active Compounds by GC-MS

The investigated extracts and fractions were dissolved to 5 mg/mL in DCM, and 200 *μ*L of the dissolved sample was transferred to a GC-MS vial, to which 50 *μ*L of TMSH was added. The mixture with a concentration of 4 mg/mL was shaken for 30 s. A GCMS-QP2010 gas chromatograph mass spectrometer was utilized (Shimadzu Scientific Instruments, Columbia, MD, USA). Helium 5.0 was used as carrier gas, and the column flow was set at a constant value of 1.7 mL/min. The column used was a Zebron ZB-5 60 m × 0.25 mm (inner diameter), 0.25 *μ*m (film thickness) (Phenomenex, Torrance, CA, USA). The software used was Lab Solutions GC-MS (version 2.50 SU3, Shimadzu). 1 *μ*L of sample was injected of the prepared solutions with a split 1 : 10, and analysis was performed in the EI mode (70 eV, 250°C ion source temperature). The injection temperature was 270°C. A method with a temperature gradient from 120°C to 320°C in 40 min and 5 min hold on 320°C was applied for analysis. A mixture of trihydroxy- and epoxy-hydroxy FAMEs from no. 1a was derivatised by BSTFA-TMCS (99 : 1) for 30 min at 50°C and detected by GC-MS in the same conditions as above. The analyses were performed on an Agilent Technologies 6890 N Network GC equipped with an Agilent Technologies 5973 inert mass selective detector and a Combi PAL autosampler (CTC Analytics). The column used was a DB-5 with dimensions of 30 m × 0.25 mm (inner diameter), 0.23 *μ*m (film thickness) (Agilent Technologies). The software used was MSD Chemstation 2004.

### 2.6. NF-*κ*B Transactivation Assay

HEK 293 cells, stably transfected with as NF-*κ*B-responsive luciferase reporter (Panomics, Fremont, CA, USA), were seeded in 10 cm dishes and transfected with 5 *μ*g pEGFP-N1 (Clontech, Mountain View, CA, USA). Six hours later, cells were transferred to 96-well plates and incubated overnight (5% CO_2_, 38°C). On the next day, the medium was exchanged with a serum-free DMEM, and cells were treated with the indicated extracts or fractions. The solvent vehicle (0.1% DMSO) served as negative control and 5 *μ*M parthenolide as positive control. After one hour, cells were stimulated with 2 ng/mL human recombinant TNF-*α* for 6 h, then the medium was removed, and cells were lysed. The luminescence of the firefly luciferase and the fluorescence of the enhanced green fluorescent protein (EGFP) were quantified on a GeniosPro plate reader (Tecan, Austria). The luciferase signal derived from the NF-*κ*B reporter was normalized with the EGFP-derived fluorescence to account for differences in the cell number.

### 2.7. PPAR Luciferase Reporter Gene Assay

The PPAR-luciferase reporter gene assay was performed as previously described [26]. HEK293 cells were transiently transfected with a PPAR*α* or PPAR*γ* receptor expression plasmid, a reporter plasmid (tk-PPREx3-luc), and a green fluorescent protein plasmid (pEGFP-N1) as an internal control. The cells were harvested 6 h after the transfection and reseeded in 96-well plates (5 × 10^4^ cells/well). In the initial tests, cells were treated with 10 *μ*g/mL of the indicated extracts. In the dose-response experiments, cells were treated with 0, 3, 27 or 81 *μ*g/mL of the DCM extracts of CHMs nos. 1a, 1b, 2a, 2b, 12a, and 12b. To account for potential effects of the solvent, 0.1% DMSO served as vehicle control. As positive controls, 50 nM GW7647 and 5 *μ*M troglitazone were used to activate PPAR*α* and PPAR*γ*, respectively. Treated cells were incubated for 18 h. After cell lysis, the luminescence of the firefly luciferase and the fluorescence of EGFP were quantified on a GeniosPro plate reader (Tecan, Salzburg, Austria). The luminescence signals were normalized to the EGFP-derived fluorescence to account for differences in cell number or transfection efficiency.

### 2.8. Statistical Analysis

Statistical analysis was performed using Prism Software (ver. 4.03; GraphPad Software Inc., San Diego, CA). Data were normalized to DMSO-treated control of which the mean value was set as 1.0. The data shown represent arithmetic mean and standard deviation of 3-4 independent experiments. Statistical significance was determined by a one-way analysis of variance combined with a Dunnett's Multiple Comparison posttest. Results with *P* < 0.05 were considered significant.

## 3. Results and Discussion

In this study, extracts of important Chinese herbal medicines from fourteen different plant species (Table 1) were tested for their potential to inhibit NF-*κ*B and/or to activate PPAR*α* and PPAR*γ*.

Extracts of CHMs of six out of fourteen plant species reduced TNF-*α*-induced NF-*κ*B activity significantly (*P* < 0.05) (Table 2). In fact, this is the first report of a significant NF-*κ*B inhibitory activity of extracts from* A. julibrissin*, *Arisaema *sp., *J. effuses*, and *P. ternata*. A moderate (see explanation accompanying Table 2) reduction of TNF-*α*-induced NF-*κ*B activity (*P* < 0.05) was found for the PE, EtOAc, and MeOH extracts of *A. julibrissin *no.1a and the EtOAc extract of no. 1b and *C. monnieri* no. 5, and PE extracts of *Arisaema *sp. no. 2a and *P. ternata *no. 12b. The EtOAc extracts of *F. suspensa* no. 7, *J. effusus *no. 8, and the aqueous extract of *A. julibrissin *no. 1b exhibited an especially strong ability (see explanation accompanying Table 2) to inhibit NF-*κ*B activity. Betulinic acid was isolated from the DCM extract of the bark of *A. julibrissin *no. 1a [27]. Many terpenoids, including betulinic acid, are reported to effectively inhibit NF-*κ*B signaling [9, 28]. However, we neither detected NF-*κ*B inhibition nor PPAR*α* and PPAR*γ* activation by betulinic acid in concentrations up to 30 *μ*M excluding betulinic acid as one of the main active principles in our cellular models. Osthole might in part have contributed to the observed activities of the EtOAc extract of *C. monnieri *no. 5 [29–31].

When testing for a PPAR*α* and PPAR*γ* agonistic activity, a significant effect (*P* < 0.05) was observed for apolar extracts of seven out of the fourteen plants used as CHM (Table 2). Moderate to strong PPAR*α* and PPAR*γ* activity was observed for apolar extracts from *A. julibrissin* no. 1a and 1b, *Arisaema *sp. no. 2a, *C. monnieri *no. 5, *P. ternata *no. 12a and *T. terrestris* no. 14 (*P* < 0.05). Apolar extracts of *Lilium *sp. no. 9 and *L. lophatherum *no. 10 activated PPAR*α* moderately (*P* < 0.05). Previously, a saponin from fruits of *T. terrestris *[32, 33] and aqueous extracts of arillus from *D. longan *and rhizomes of *P. ternata* [34–36] were reported to increase PPAR*α* and PPAR*γ* gene expression levels.

**Figure 1:**
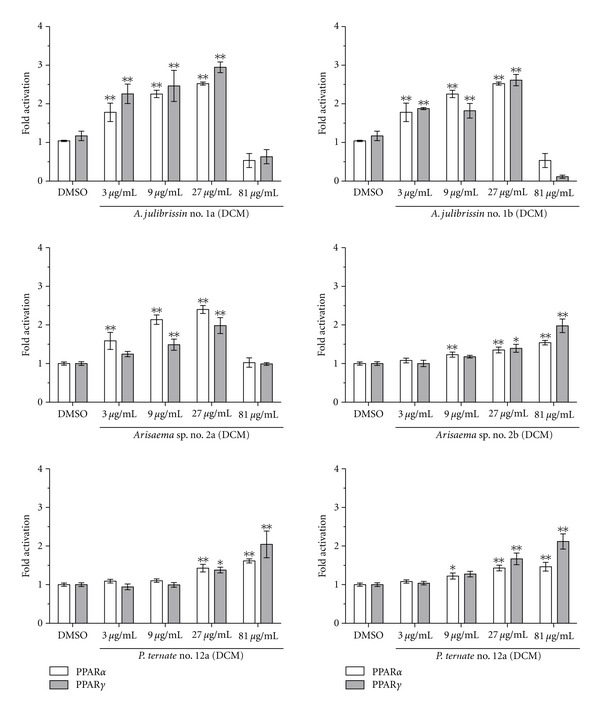
Dose-response experiments with DCM extracts of CHMs of *A. julibrissin *no. 1a and b, *Arisaema *sp. no. 2a and b, and *P. ternata* no. 12a and b. HEK293 cells were transiently transfected with the expression plasmid for PPAR*α* or PPAR*γ*, the reporter plasmid pPPRE-tk3x-luc, and the internal control plasmid (EGFP). The transiently transfected cells were incubated for 18 h with 3, 9, 27, and 81 *μ*g/mL of each indicated extract. Furthermore, the cells were similarly incubated with 0.1% DMSO (negative control), 50 nM GW7647 (PPAR*α* agonist, activated PPAR*α* 2.5–3.5 fold but not PPAR*γ*), or 5 *μ*M troglitazone (PPAR*γ* agonist, activated 3.5–6.5 fold PPAR*γ* but not PPAR*α*) as positive controls (not shown). Luciferase activity and fluorescence intensity were measured. Results are presented as mean ± SD, (*n* = 4). Significantly different from the negative control (ANOVA), *P < 0.05; **P < 0.01.

 A selection of CHMs with strong activity, namely, *A. julibrissin* no. 1a and b, *Arisaema *sp. no. 2a and b and of *P. ternata *no. 12a, and b were further studied. The DCM extracts of no. 1a, 1b, 2a, 2b, 12a, and 12b showed a significant dose-dependent increase of PPAR*α* and PPAR*γ* activity (*P* < 0.01) (Figure 1), whereby those of nos. 1a and 1b from *A. julibrissin *and no. 2a from *Arisaema *sp. were most potent. Loss of activity at 81 *μ*g/mL of these extracts was possibly due to toxicity or unspecific inhibitory action.

**Figure 2:**
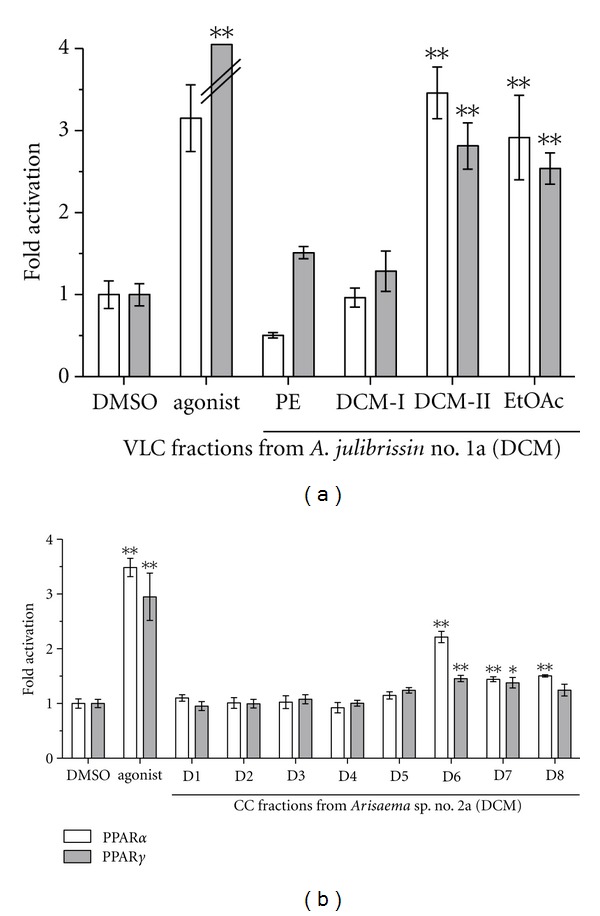
Comparison of the PPAR*α*- and PPAR*γ*-activating potential of the VLC fractions petroleum ether (PE), DCM-I and -II, and ethyl acetate extract (EtOAc) of the DCM extract from *A. julibrissin *no. 1a (a) and the DCM fractions D1-8 from *Arisaema *sp. no. 2a. (b). HEK293 cells, transiently transfected with expression plasmids for PPAR*α* or PPAR*γ*, luciferase reporter (tk-PPREx3-luc), and EGFP as internal control, were incubated for 18 h with 10 *μ*g/mL of the indicated fraction, solvent vehicle (0.1% DMSO, negative control), and 50 nM GW7647 (PPAR*α* agonist) or 5 *μ*M troglitazone (PPAR*γ* agonist) as positive control. Luciferase activity and fluorescence intensity were measured. Results are presented as mean ± SD (*n* = 4). Significantly different from the negative control (ANOVA), *P < 0.05; **P < 0.01.

**Figure 3:**
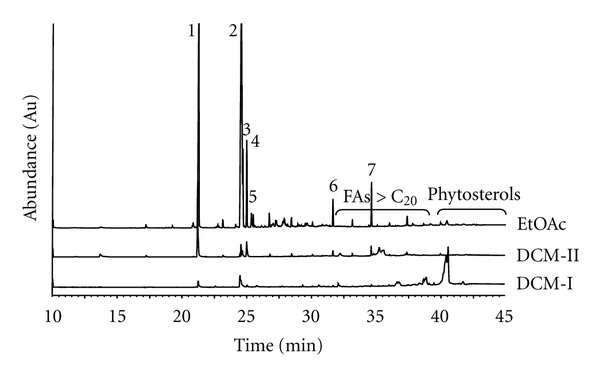
Comparative analysis by GC-MS of the DCM-I and -II and EtOAc VLC fractions (1 *μ*L of 4 mg/mL in DCM) from the mother DCM extract of CHMs nos. 1a of *A. julibrissin*. FAMEs formed from the present FAs after derivatisation by TMSH were detected by GC-MS on a Zebron ZB-5 column (60 m × 0.25 mm, 0.25 *μ*m) (Phenomenex) with a temperature gradient of 120°C-320°C in 40 min and 5 min hold; carrier gas: helium; flow rate: 1.7 mL/min. Ion source: EI 70 eV, 250°C. The following FAs were identified by GC-MS with a relative content (%) in each enriched extract given in the order DCMI; DCMII and EtOAc: (1) palmitic acid (2.9; 37; 23%), (2) linoleic acid (4.5; 1.0; 47%), (3) 9-octadecenoic acid (3.3; 5.1; 4.4%), (4) oleic acid (—; 3.8; 5.8%), (5) octadecanoic acid (3.3; 9.4; 2.2%), (6) docosanoic acid (—; 4.5; 1.7%), and (7) tetracosanoic acid (—; 2.3; 3.0%).

**Figure 4:**
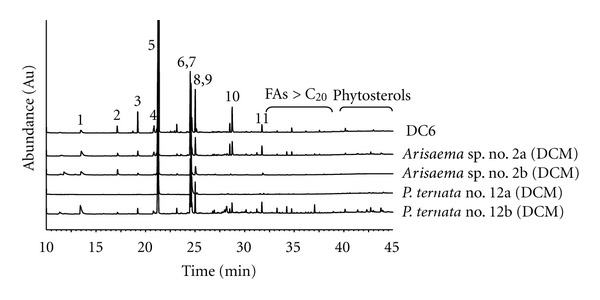
Comparative analysis by GC-MS of fraction D6 from the DCM extract of the CHM no. 2a from *Arisaema *sp. with DCM extracts from CHMs no. 2a and b of *Arisaema *sp. and no. 12a and b from *P. ternata* (1 *μ*L of 4 mg/mL in DCM). FAMEs formed from the present FAs after derivatisation by TMSH were detected by GC-MS as in Figure 3. The following FAs were identified by GC-MS with a relative content (%) in each enriched extract given in the order of D6, CHM no. 2a, 2b, 12a and 12b: (1) nonanedioic acid (1.1; 4.1; 3.4;—; 3.8%), (2) tetradecanoic acid (1.0; 2.1; 1.0; —; 0.4%), (3) pentadecanoic acid (2.4; 1.5; 0.8;—; 0.9), (4) 9-hexadecenoic acid (1.5; 1.7; 0.4;—; 0.1%), (5) palmitic acid (61; 39; 74; 30; 37%) (6) linoleic acid (6.5; 8.9; 0.5; 36; 6.6%), (7) 9-octadecenoic acid (4.2; 8.2; 4.6; 17; 9.2%), (8) oleic acid (2.9; 1.6; 1.0;—; 2.6%), (9) octadecanoic acid (8.5; 4.5; 3.4; 2.6; 4.4%), (10) 13-phenyltridecanoic acid (3.4; 3.9; 0.5; 0.1; 2.6%), and (11) docosanoic acid (0.2; 0.9; 0.7;—; 0.3).

 Bioassay-guided fractionation showed that the fraction “DCM-II” (see Section 2) of the DCM extract from *A. julibrissin *no. 1a significantly activated PPAR*α* by 3.5-fold (±0.31, *P* < 0.01) and PPAR*γ* by 2.8-fold (±0.28, *P* < 0.01) (compared to DMSO as solvent control). The EtOAc VLC fraction significantly activated PPAR*α* by 2.9-fold (±0.52, *P* < 0.01) and PPAR*γ* by 2.5-fold (±0.19, *P* < 0.01). The apolar fraction “D6” from *Arisaema *sp. no. 2a significantly activated PPAR*α* by 2.2-fold (±0.10, *P* < 0.01) and PPAR*γ* by 1.5-fold (±0.06, *P* < 0.01) (Figure 2). GC-MS analysis after derivatisation revealed that the VLC fractions of *A. julibrissin* no. 1a (DCM) and fraction “D6” from *Arisaema *sp. no. 2a contained mainly fatty acids (FAs), such as palmitic acid (16 : 0), stearic acid (18 : 0), and linoleic acid (18 : 2) (Figures 3 and 4) that are known as PPAR agonists [11]. 13-Phenyltridecanoic acid (13 : 0) was found as a compound specific for aroids [37]. Especially polyunsaturated FAs activate PPARs potently [38]. Wolfrum et al. reported palmitic acid (18 : 0), linoleic acid (18 : 2), oleic acid (18 : 1), and stearic acid (18 : 0) to activate PPAR*α* by 2.5-, 4.6-, 3.5-, and 2-fold, respectively [39]. The processing might have influenced availability of FA from rhizomes of *P. ternata*. The concentration of FAs detected was higher in the DCM extract of processed rhizomes of *P. ternata *(no. 12b) than in that of the crude rhizomes (no. 12a) (Figure 4). This was in line with the observation that the DCM extract of the processed 12b was PPAR*α* and PPAR*γ* active at slightly lower concentration than that of the crude rhizomes (no. 12a) (Figure 1). The activation of PPAR*α* by fraction “D6” may be attributed largely to palmitic acid, which is highly enriched in fraction “D6” reaching 61% of the total content (Figure 4). *α*-Spinasterol-3-*O*-*β*-D-glucopyranoside from *A. julibrissin *and cerebrosides isolated from the DCM extract of rhizomes of *Arisaema *sp. and *P. ternata* were tested; however, did not show agonistic activity towards PPAR*α*- and PPAR*γ*-activity *in vitro*.

It became further evident that the content of the FAs present in “D6” was also high in the DCM extracts from CHMs from *Arisaema *sp. and *P. ternata* (Figure 4), especially of palmitic acid (30%–74%). The CHMs of *T. terrestris *and *C. monnieri* were previously described to contain FAs including palmitic acid, stearic acid, oleic acid, and linoleic acid [19, 32]. Besides common FAs, trihydroxy- and epoxy-hydroxy FAMEs and *α*-spinasterol-3-*O*-*β*-D-glucopyranoside were isolated from the MeOH extract of *A. julibrissin *no. 1a. Although mono- and dihydroxylated FAs are natural PPAR*γ* agonists [40], trihydroxy- and epoxy-hydroxy FAMEs as well as their FA derivatives, identified by GC-MS [21], lacked PPAR*α* and PPAR*γ* activity *in vitro *in this study. Interestingly, chronic exposure to high levels of palmitic acid and stearic acid, identified as one of the main active principles in this study, are associated with lipotoxicity or insulin resistance, the main causative factor of type 2 diabetes and the metabolic syndrome [33, 39]. However, unsaturated free FAs, such as linoleic acid and oleic acid, do not promote insulin resistance [41]. In contrast, even a protective effect of oleic acid against palmitate-induced insulin resistance in L6 rat myotubes has been demonstrated [42]. Moreover, FAs have recently been identified as rather potent inhibitors of PTP1B, the main negative regulator of insulin and leptin signaling [43]. Thus, the impact of FAs on health and disease seems to highly depend on duration of exposure, concentration of the FA, or the degree of saturation.

## 4. Conclusion

In this study, extracts from CHMs were tested for a putative PPAR*α*- or PPAR*γ*-activating and NF-*κ*B-inhibiting effect. Out of the fourteen plant species of which extracts were tested, 43% exhibited NF-*κ*B inhibitory and 50% PPAR activating effects. The apolar PPAR active extracts and enriched fractions from flos and cortex of *A. julibrissin* and rhizomes of *Arisaema *sp. and *P. ternata* mainly contained fatty acids as PPAR-agonists, including palmitic acid, linoleic acid, oleic acid, and stearic acid. The outcome of this study contributes to the molecular understanding and explanation of some effects elicited by extracts of these three traditional Chinese herbs by revealing PPAR activation due to the present fatty acids.

## Supplementary Material

Workflow of bioassayguided fractionation and isolation.

## Figures and Tables

**Table 1:** The plant species and the respective Chinese herbal medicines tested for potential activation of human PPAR*α* and PPAR*γ* and inhibition of TNF-*α*-induced NF-*κ*B activity.

Plant species	Family	Plant part	Pin Yin	CHM no.
*Albizia julibrissin* Durazz.	Fabaceae	Cortex	hé huān pí	1a
		Flos	hé huān huā	1b
*Arisaema* sp.	Araceae	Rhizoma*	zhì tiān nán xīng	2a
		Rhizoma**	zhì tiān nán xīng	2b
*Arnebia/Lithospermum *sp.	Boraginaceae	Radix	z*ǐ* cǎo	3
*Atractylodes macrocephala* Koidz.	Asteraceae	Rhizoma	bái zhú	4
*Cnidium monnieri* (L.) Cuss.	Apiaceae	Fructus	shé chuáng z*ǐ*	5
*Dimocarpus (Euphoria) longan* Lour.	Sapindaceae	Arillus	long yǎn ròu	6
*Forsythia suspensa* (Thunb.) Vahl.	Oleaceae	Fructus	lián qiào	7
*Juncus effusus *L.	Juncaceae	Medulla	d*ē*ng xīn cǎo	8
*Lilium *sp.	Liliaceae	Bulbus	bǎi hé	9
*Lophatherum gracile* Brongn.	Poaceae	Herba	dàn zhú yè	10
*Nelumbo nucifera* Gaertn.	Nelumbonaceae	Plumula	lián z*ǐ* xīn	11
*Pinellia ternata* (Thunb.) Breit.	Araceae	Rhizoma	bàn xià	12a
		Rhizoma**	zhì bàn xià	12b
*Polygonum multiflorum* Thunb.	Polygonaceae	Caulis	sh*ŏ*u wū téng	13
*Tribulus terrestris* L.	Zygophyllaceae	Fructus	cì jí lí	14

Processed by cooking * or soaking ** in water with alum (KAl(SO_4_)_2_·12H_2_O).

**Table 2:** Screening of Chinese herbal medicines for agonistic activity towards PPAR*α* and PPAR*γ* and inhibition of TNF-*α*-induced NF-*κ*B activity *in vitro*. PE = petroleum ether, EtOAc = ethyl acetate, MeOH = methanol. Dry material from all test samples was dissolved in DMSO at a concentration of 10 mg/mL and tested at a final concentration of 10 *μ*g/mL. Regarding PPARs: no activation compared to control (0.1% DMSO): −; 1.5- to 2-fold activity compared to control (*P* < 0.05): +; more than 2-fold of the control (*P* < 0.05): ++. Regarding NF-*κ*B: inhibition in the range of 0–50% compared to the vehicle-treated control (0.1% DMSO): −; 50–80% inhibition (*P* < 0.05): +; over 80% inhibition (*P* < 0.05): ++. At least three independent experiments were performed with every sample, and statistical analysis was performed by ANOVA.

Plant species	CHM no.	Extract	PPAR*α*	PPAR*γ*	NF-*κ*B
			activation	activation	inhibition
*Albizia julibrissin *Durazz.	1a	PE	++	++	+
		EtOAc	++	++	+
		MeOH	+	+	+
		H_2_O	−	−	+
	1b	PE	+	+	−
		EtOAc	++	++	+
		MeOH	−	−	−
		H_2_O	−	−	++

*Arisaema* sp.	2a	PE	++	+	+
		EtOAc	−	−	−
		MeOH	−	−	−
		H_2_O	−	−	−

*Arnebia/Lithospermum *sp.	3	PE	−	−	−
		EtOAc	−	−	−
		MeOH	−	−	−
		H_2_O	−	−	−

*Atractylodes macrocephala* Koidz.	4	PE	−	−	−
		EtOAc	−	−	−
		MeOH	−	−	−
		H_2_O	−	−	−

*Cnidium monnieri* (L.) Cuss.	5	PE	−	−	−
		EtOAc	++	+	+
		MeOH	−	−	−
		H_2_O	−	−	−

*Dimocarpus (Euphoria) longan* Lour.	6	PE	−	−	−
		EtOAc	−	−	−
		MeOH	−	−	−
		H_2_O	−	−	−

*Forsythia suspensa* (Thunb.) Vahl.	7	PE	−	−	+
		EtOAc	−	−	++
		MeOH	−	−	−
		H_2_O	−	−	−

*Juncus effusus *L.	8	EtOAc	−	−	++
		MeOH	−	−	+
		H_2_O	−	−	−

*Lilium *sp.	9	EtOAc	+	−	−
		MeOH	−	−	−
		H_2_O	−	−	−

*Lophatherum gracile* Brongn.	10	PE	+	−	−
		EtOAc	+	−	−
		MeOH	−	−	−
		H_2_O	−	−	−

*Nelumbo nucifera* Gaertn.	11	PE	−	−	−
		EtOAc	−	−	−
		MeOH	−	−	−
		H_2_O	−	−	−

*Pinellia ternata* (Thunb.) Breit.	12b	PE	++	+	+
		EtOAc	−	−	−
		MeOH	−	−	−
		H_2_O	−	−	−

*Polygonum multiflorum* Thunb.	13	PE	−	−	−
		EtOAc	−	−	−
		MeOH	−	−	−
		H_2_O	−	−	−

*Tribulus terrestris* L.	14	PE	−	−	−
		EtOAc	+	+	−
		MeOH	−	−	−
		H_2_O	−	−	−

## Abbreviations

ASE: Accelerated solvent extractor
BSTFA-TMCS: *N,O*-bis(trimethylsilyl) trifluoroacetamide (BSTFA) and trimethylchlorosilane
CC: Column chromatography
CHM: Chinese herbal medicine
DCM: Dichloromethane
DMEM: Dulbecco's modified Eagle's medium
DMSO: Dimethylsulfoxide
EGFP: Enhanced green fluorescent protein
EI MS: Electron ionization mass spectrometry
EtOAc: Ethylacetate
FA: Fatty acid
FAME: Fatty acid methyl ester
FBS: Fetal bovine serum
GC-MS: Gas chromatography mass spectrometry
LPS: Lipopolysaccharide
MeOH: Methanol
NF-*κ*B: Nuclear factor *κ*B
NMR: Nuclear magnetic spin resonance
PE: Petroleum ether
PPAR: Peroxisome proliferator-activated receptor
TNF: Tumor necrosis factor
TMSH: Trimethylsulfoniumhydroxide
VLC: Vacuum liquid chromatography.
