# Supplementary material for: Selected Extracts of Chinese Herbal Medicines: Their Effect on NF-κB, PPARα and PPARγ and the Respective Bioactive Compounds
Source: Evid Based Complement Alternat Med. 2012 May 23;2012:983023. doi: 10.1155/2012/983023 (PMC3366346; doi:10.1155/2012/983023)
Supplement: Supplementary file 1 — Workflow of bioassayguided fractionation and isolation. [file 983023.f1.doc]

**
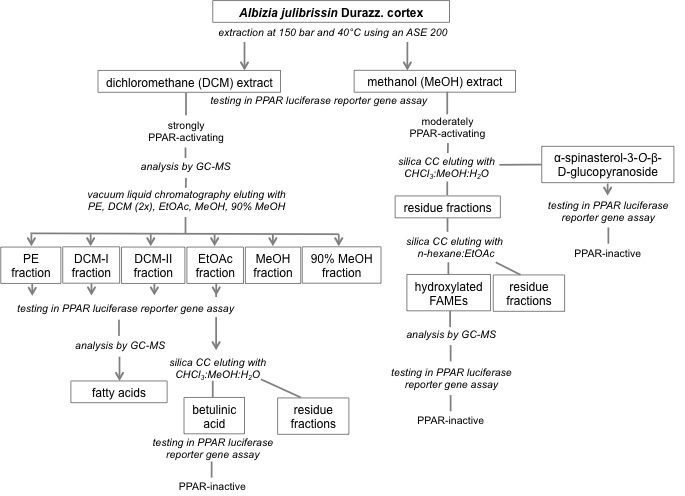
**

**Scheme 1**. Bioassay-guided fractionation-isolation scheme of cortex of *A. julibrissin* (Fabaceae). ASE = accelerated solvent extractor, PE = petroleum ether, DCM = dichloromethane, EtOAc = ethylacetate, MeOH = methanol, CC = column chromatography, FAME = fatty acid methyl ester.

**
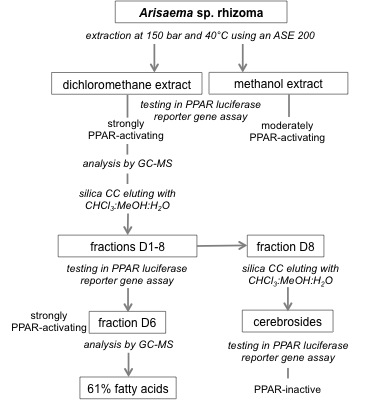
**

**Scheme 2**. Bioassay-guided fractionation-isolation scheme of rhizomes of *Arisaema* sp. (Araceae). ASE = accelerated solvent extractor, CC = column chromatography.
